# Multiple Novel Traits without Immediate Benefits Originate in Bacteria Evolving on Single Antibiotics

**DOI:** 10.1093/molbev/msab341

**Published:** 2021-12-03

**Authors:** Shraddha Karve, Andreas Wagner

**Affiliations:** 1 Department of Evolutionary Biology and Environmental Studies, University of Zurich, Zurich, Switzerland; 2 Swiss Institute of Bioinformatics, Quartier Sorge-Batiment Genopode, Lausanne, Switzerland; 3 The Santa Fe Institute, Santa Fe, NM, USA; 4 Stellenbosch Institute for Advanced Study (STIAS), Wallenberg Research Centre, Stellenbosch University, Stellenbosch, South Africa

**Keywords:** novel traits, antimicrobials, pleiotropy, experimental evolution

## Abstract

How new traits originate in evolution is a fundamental question of evolutionary biology. When such traits arise, they can either be immediately beneficial in their environment of origin, or they may become beneficial only in a future environment. Compared to immediately beneficial novel traits, novel traits without immediate benefits remain poorly studied. Here we use experimental evolution to study novel traits that are not immediately beneficial but that allow bacteria to survive in new environments. Specifically, we evolved multiple *E. coli* populations in five antibiotics with different mechanisms of action, and then determined their ability to grow in more than 200 environments that are different from the environment in which they evolved. Our populations evolved viability in multiple environments that contain not just clinically relevant antibiotics, but a broad range of antimicrobial molecules, such as surfactants, organic and inorganic salts, nucleotide analogues and pyridine derivatives. Genome sequencing of multiple evolved clones shows that pleiotropic mutations are important for the origin of these novel traits. Our experiments, which lasted fewer than 250 generations, demonstrate that evolution can readily create an enormous reservoir of latent traits in microbial populations. These traits can facilitate adaptive evolution in a changing world.

## Introduction

How novel traits evolve is a central question in evolutionary biology (Bock 1959; Pigliucci 2008; Wagner and Lynch 2010; Wagner 2011, Erwin 2021). Especially, important for Darwinian evolution are novel traits that are beneficial in a present or future environment, because they increase fitness or establish a new ecological niche (Hochberg et al. 2017). Some novel traits comprise new and beneficial structures, such as wings in birds (Wagner and Lynch 2010). Others are purely physiological, such as the ability to utilize a new carbon source in bacteria (Blount et al. 2008). 

Microorganisms are highly suitable to study novel physiological traits, because their large population sizes and short generation times can make the evolutionary origin of such traits observable on laboratory time scales. In addition, microbial genomes are small, and genomic changes that bring forth novel traits can be easily studied by genome sequencing (Bennett and Hughes 2009; Toll-Riera et al. 2016).

A novel trait may or may not provide a benefit in the environment where it originates. If a novel trait provides an immediate benefit, natural selection can drive its spread through a population. Mutations can further refine the trait during this process. Some novel traits with immediate benefits can evolve quite rapidly in laboratory evolution experiments. For example, during experimental evolution in chemically minimal environments containing only a single carbon source, *Pseudomonas aeruginosa* populations evolved multiple novel metabolic traits within a few hundred generations (Toll-Riera et al. 2016). Other novel traits with immediate benefit may require much longer to originate. A case in point is an ongoing long-term evolution experiment with *Escherichiacoli*, which is carried out in a growth medium that contains citrate as a chelating agent. Unlike many bacteria, *E. coli* cannot grow aerobically on citrate. Although this ability did eventually evolve, it did so only once among 12 populations, and only after 31,000 generations (Blount et al. 2008, 2012).

A novel trait may not be beneficial in the environment in which it originates. A trait like this is only *potentially* beneficial. It can emerge in at least two different ways. First, the trait can be a by-product of the adaptive evolution of other traits. That is, a DNA mutation that results in a beneficial trait in the environment of origin can give rise to another trait that is beneficial only in a different environment. Second, a novel trait can be caused by one or more neutral or mildly deleterious mutations that can persist in a population because of genetic drift. The trait can then become beneficial when the environment changes.

Even though potentially beneficial traits can be difficult to identify, several physiological novel traits without immediate benefits have been discovered both in the wild and in the laboratory (Dantas et al. 2008; Meijnen et al. 2008; Leiby and Marx 2014; Karve et al. 2015; Toll-Riera et al. 2016). For instance, soil microbes from pristine habitats can metabolize the synthetic antibiotic ciprofloxacin, which they are unlikely to have encountered in the wild (Dantas et al. 2008). In a long-term evolution experiment with *E.coli*, cells improved their viability on carbon sources that they were unlikely to have encountered during the experiment (Leiby and Marx 2014). In another evolution experiment, *E. coli* improved its growth on high concentrations of the heavy metal cobalt in an environment lacking cobalt (Karve et al. 2015). A strain of *Pseudomonas putida* acquired the ability to metabilize arabinose while evolving in a xylose-containing medium devoid of arabinose (Meijnen et al. 2008). In addition, computational studies have predicted that metabolic novel traits without immediate benefits may be frequent in the carbon metabolism of *E. coli* (Barve and Wagner 2013; Hosseini and Wagner 2016). Beyond such computational predictions and individual examples, we know little about the incidence of novel traits that are not immediately beneficial. In particular, we do not know whether they originate frequently or rarely. We also do not know whether they require many or complex genomic changes.

To help answer both questions, we performed evolution experiments in *E. coli*. Briefly, we evolved multiple replicate populations of *E. coli* in five environments, each of which contained a different antibiotic, and then examined the capacity of evolved clones from these populations to grow in 236 other environments. We chose antibiotic-containing environments for several reasons. First, both the mechanism of action of multiple antibiotics and the process of evolutionary adaptation to these antibiotics are well-studied, with a wealth of information about adaptive genetic changes and their phenotypes. Second, we reasoned that our results may be relevant for the clinic, if the evolution of antibiotic resistance frequently helps bacteria survive in other stressful environments. Third, because bacteria encounter antibiotics in the wild, our observations about the incidence of novel traits without immediate benefits may also be relevant to wild populations.

Some previous work has examined how adaptive resistance to one antibiotic can cause “collateral” resistance (or sensitivity) to other antibiotics (Imamovic and Sommer 2013; Lázár et al. 2014; Podnecky et al. 2018). However, these studies focused on a few clinically relevant antibiotics. In contrast, the 236 environments we study are much more diverse. 164 of the environments contain nonantibiotic growth-inhibiting substances that include detergents, oxidants, surfactants, organic, and inorganic salts as well as pyridine analogs. Viability in many of these environments readily emerged during our short evolution experiment. Whole-genome sequencing identified only a few mutations in each evolved clone, which shows that the novel traits we identified did not require many or complex genome changes. Our sequence data also suggest that antibiotic resistance mutations have pleiotropic effects that facilitate survival in multiple environments.

## Results

### Experimental Design

**Fig. 1.**
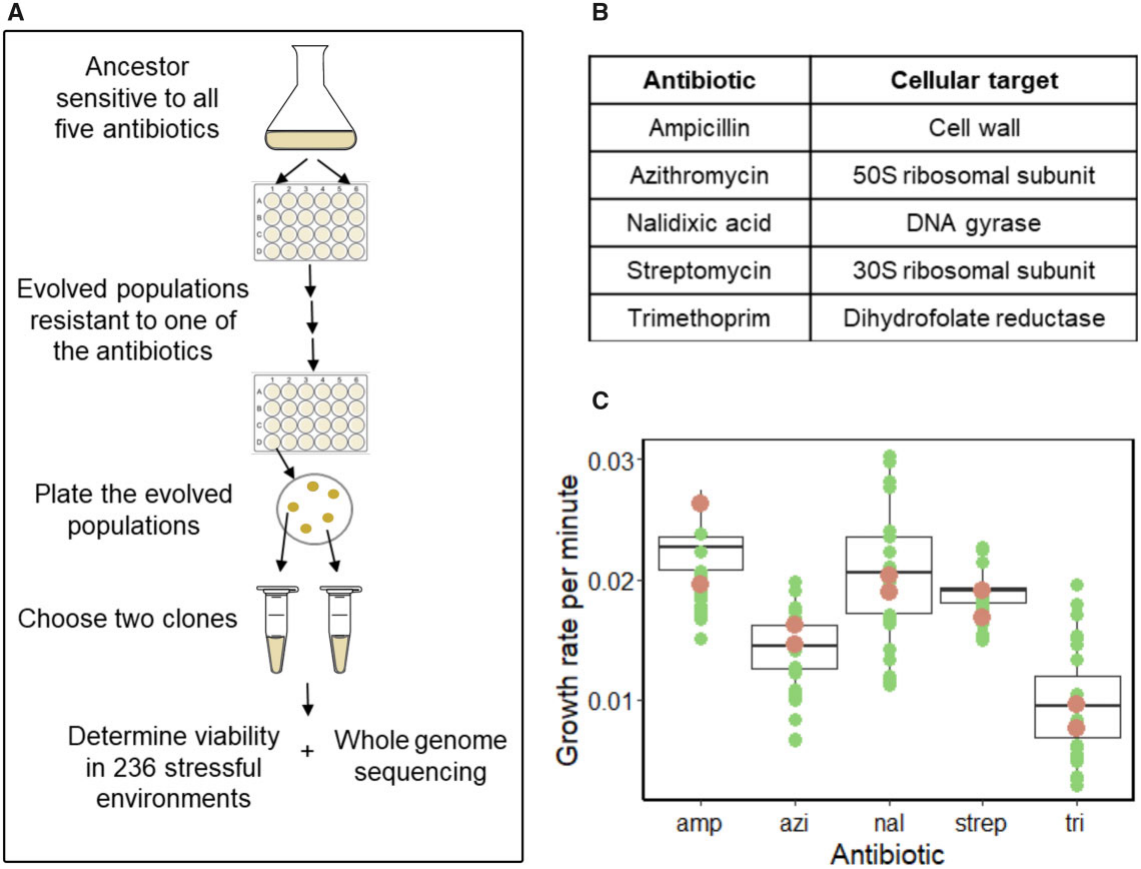
Overview of experimental evolution. (*A*) We performed five independent evolution experiments, each in an environment containing one of five antibiotics, each of which has a different target and mode of action (*B*). In each experiment we evolved eight replicate *Escherichia coli* populations in parallel for ∼100–200 generations (see supplementary table S1, Supplementary Material online for details). At the end of the evolution experiment, all populations could grow at the IC_90_ of the ancestor in the respective antibiotic. We chose two representative clones from every antibiotic environment to identify novel traits on Biolog phenotyping microarrays, and to sequence their genomes. (*C*) Growth rates of eight replicate populations (black boxes, vertical axis) on each of the five antibiotics (horizontal axis) on which they evolved, at the end of experimental evolution. In each box plot, the thick horizontal line represents the mean growth rate of the eight evolved populations, and the lower and upper boundaries of the box represent the first and the third quantile, respectively. Whiskers show 95% CIs. Circles show the growth rates of 24 clones (three randomly chosen clones from each of the eight populations). Orange circles represent the growth rates of the 2 clones, out of 24, that we selected for novel trait assays and for whole-genome sequencing. We estimated all growth rates at the IC_90_ of the respective antibiotic for all populations and clones (supplementary table S2, Supplementary Material online provides doubling times corresponding to estimated growth rates).

We performed five independent laboratory evolution experiments, each with eight replicate *E. coli* populations (fig. 1*A*). The five experiments differed in the antibiotic that cells were exposed to during evolution. These antibiotics were ampicillin (amp), azithromycin (azi), nalidixic acid (nal), streptomycin (strep), and trimethoprim (tri). We chose these five antibiotics because they have very different modes of actions and cellular targets (Blair et al. 2015; Kapoor et al. 2017; fig. 1*B*). Specifically, ampicillin is a β-lactam antibiotic that targets bacterial cell-wall synthesis. Azithromycin is a macrolide antibiotic that interferes with protein synthesis by binding to the 50S subunit of the ribosome. Nalidixic acid is a quinolone antibiotic that inhibits the activity of DNA gyrase, an enzyme that is essential for DNA synthesis. Streptomycin is an aminoglycoside antibiotic that binds to the 16srRNA of the 30S ribosomal subunit and inhibits protein synthesis. Trimethoprim inhibits the activity of the enzyme dihydrofolate reductase, which is necessary for the synthesis of thymidine, one of the four DNA bases.

We evolved each population in a single well of a 24-well plate that contained 2 ml of Luria Bertani (LB) medium supplemented with the respective antibiotic, which we also refer to as the *evolution environment*. We transferred 4 μl of culture volume to fresh medium daily to propagate the population, and allowed each population to evolve until it was able to grow at the IC_90_ of the ancestral strain, that is, at the concentration of the respective antibiotic that kills 90% of all cells in the ancestral strain (Imamovic and Sommer 2013; see Materials and Methods and supplementary Table S1, Supplementary Material online). Depending on the antibiotic, evolution required between 100 and 200 generations (12–24 days, supplementary Table S1, Supplementary Material online).

At the end of experimental evolution, we chose two evolved clones from each antibiotic environment as representatives of the evolved populations for further analyses (see Materials and Methods, black circles in fig. 1*C*). For this purpose, we plated the eight evolved populations on LB agar plates without antibiotic, and randomly chose three clones from each evolved population, that is, 24 clones for every antibiotic. We assayed the growth of these 24 clones and the eight whole populations from which we had chosen these clones at the IC_90_ of that antibiotic. We then selected two clones whose growth rates fell within the 95% confidence intervals (CIs) of the mean growth rate of the eight populations (fig. 1*C* and see Materials and Methods). In this way, we obtained two representative-evolved clones for every antibiotic environment, for a total of ten clones. For every antibiotic, except streptomycin, the two chosen clones belonged to two different replicate populations. We henceforth refer to these clones as evolved clones. In addition, we randomly chose two ancestral clones from an LB agar plate without antibiotic.

To identify novel traits, we measured the growth of both evolved and ancestral clones in 236 inhibitory environments using Biolog Phenotypic Microarrays (PM11-20, Biolog, USA; Bochner et al. 2001). We refer to these environments also as *phenotyping environments*. Each such microarray is a 96-well plate in which any one well contains one specific antimicrobial molecule in a rich growth medium, as well as a tetrazolium dye that helps quantify cellular respiration and growth.

Taken together, the arrays harbor 236 different antimicrobials. Each antimicrobial occurs in four different wells at four different concentrations, and we only used the well with the highest concentration to identify novel traits (see Materials and Methods). We call a trait novel if *both* clones that had evolved on the same antibiotic were able to survive and grow in a given phenotyping environment, even though *neither* ancestral clone could survive and grow. We call such clones *viable* in that environment. For instance, ancestral clones cannot grow at high concentrations of the iron chelator 2,2-dipyridyl (Liu et al. 2010) but clones evolved in streptomycin can. Streptomycin clones have thus evolved viability on 2,2-dipyridyl. To identify the genetic basis of each novel trait, we sequenced the genomes of all evolved and ancestral clones to at least 30-fold coverage using Illumina HiSeq (Illumina, CA, USA and see Materials and Methods).

### Novel Traits Rapidly Evolve in Environments That Do Not Directly Select for Them

**Fig. 2.**
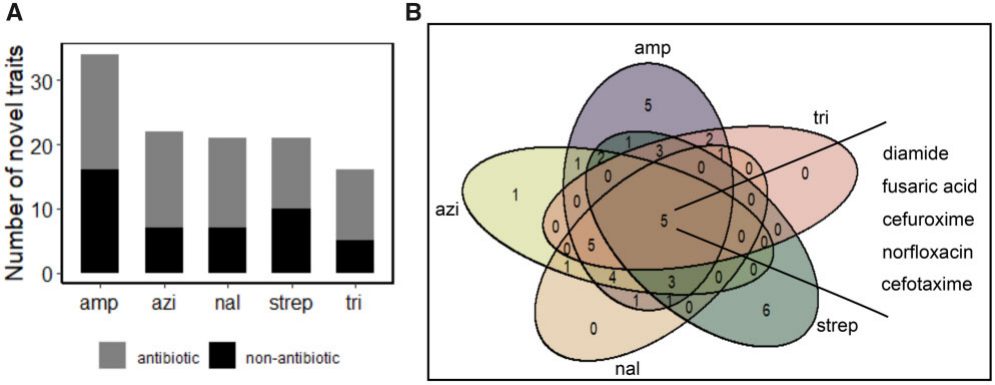
Multiple novel traits emerge in each antibiotic-containing environment. (*A*) Number of novel traits without immediate benefits (vertical axis) after experimental evolution in each of the five antibiotics (horizontal axis). We defined viability in a specific phenotyping environment as novel if *both* of the clones evolved in the same evolution environment were viable in it, even though *none* of the two ancestral clones had been viable in the phenotyping environment (see Materials and Methods). Novel traits were manifest both in phenotyping environments that contained an antibiotic (gray), and in phenotyping environments that contained a nonantibiotic antimicrobial (black). (*B*) Shared and unique novel traits among the clones evolved in different antibiotics. All evolved clones acquired viability in five environments, namely diamide, fusaric acid, cefuroxime, norfloxacin, and cefotaxime.

Between 16 and 34 novel traits evolved among the replicate populations in each of the 5 evolution experiments (fig. 2*A*). Viability evolved in a total of 42 environments: 24 that contained antibiotics and 18 that contained nonantibiotic antimicrobials (for a list of all evolved novel traits see supplementary Table S3, Supplementary Material online). Some evolved novel traits were shared between clones evolved in different antibiotics. Specifically, six, ten, nine, and five novel traits were shared between evolved clones from two, three, four, and all five evolution environments, respectively. Conversely, 12 novel traits evolved in only a single evolution environment (fig. 2*B*).

Many evolved novel traits included viability on nonantibiotic stressors with diverse mechanisms of antimicrobial action. Some of these stressors affect membrane function. They include 1-hydroxy 2-pyridine thione (Chandler and Segel 1978; Bromberger et al. 2020), gallic acid (Borges et al. 2013), and cinnamic acid (Cai et al. 2019). Others chelate intracellular iron, for example, 2,2-dipyridyl (Liu et al. 2010) and lawsone (Song et al. 2020). Lawsone additionally also causes oxidative stress and disrupts membrane potential (Song et al. 2020). The mechanism of antibacterial action for some nonantibiotic stressors is unknown. These include harmane, gallic acid, and chloroxine (5,7-dichloro 8-hydroxy quinolone). All evolved clones we examined had become viable in five phenotyping environments. Two of these contained the nonantibiotic stressors diamide and fusaric acid (fig. 2*B*). Fusaric acid is a pyridine that chelates intracellular metal ions (Bochner et al. 1980), and can also affect other important cellular functions, such as DNA synthesis and quorum sensing (Kim et al. 2021). Diamide induces oxidative stress by creating non-native disulfide bonds within and between redox-sensitive proteins. Such disulfide bonds can disrupt the redox balance of proteins, and affect important cellular processes like signal transduction and gene expression (Zehavi-Willner et al. 1970; Cumming et al. 2004; Pöther et al. 2009).

The novel traits we identified also included viability on clinically relevant antibiotics with diverse mechanisms of action including cloxacillin, ciprofloxacin, enoxacin, lomefloxacin, minocycline, nafcillin, ofloxacin, vancomycin, norfloxacin, and novobiocin. All evolved clones became viable on the antibiotics cefuroxime and cefotaxime (fig. 2*B*), which are cephalosporine β-lactam antibiotics that affect cell-wall synthesis (Källman et al. 2009; Kapoor et al. 2017). In addition, all evolved clones became viable on norfloxacin, which targets DNA gyrase (Kapoor et al. 2017; fig. 2*B*). Viability on the veterinary antibiotic tylosin originated in all populations except populations evolved on trimethoprim (supplementary Table S3, Supplementary Material online).

In sum, these observations show that novel traits evolve readily in environments that do not directly select for them. This conclusion is robust to variation in the growth threshold that we used to determine viability (supplementary fig. S4, Supplementary Material online).

### Most Antimicrobials to Which Viability Emerges Have a Different Mechanism of Action Than the Antibiotic in the Evolution Environment

Evolution of resistance to an antibiotic with a particular mode of action may lead to collateral resistance to other inhibitors with a similar mode of action. For example, bacteria that evolve in the presence of the antibiotic ampicillin, which is a β-lactam antibiotic, can evolve resistance through elevated secretion of a β-lactamase. This resistance mechanism can result in viability on cefotaxime, which is also a β-lactam antibiotic (Källman et al. 2009).

**Fig. 3.**
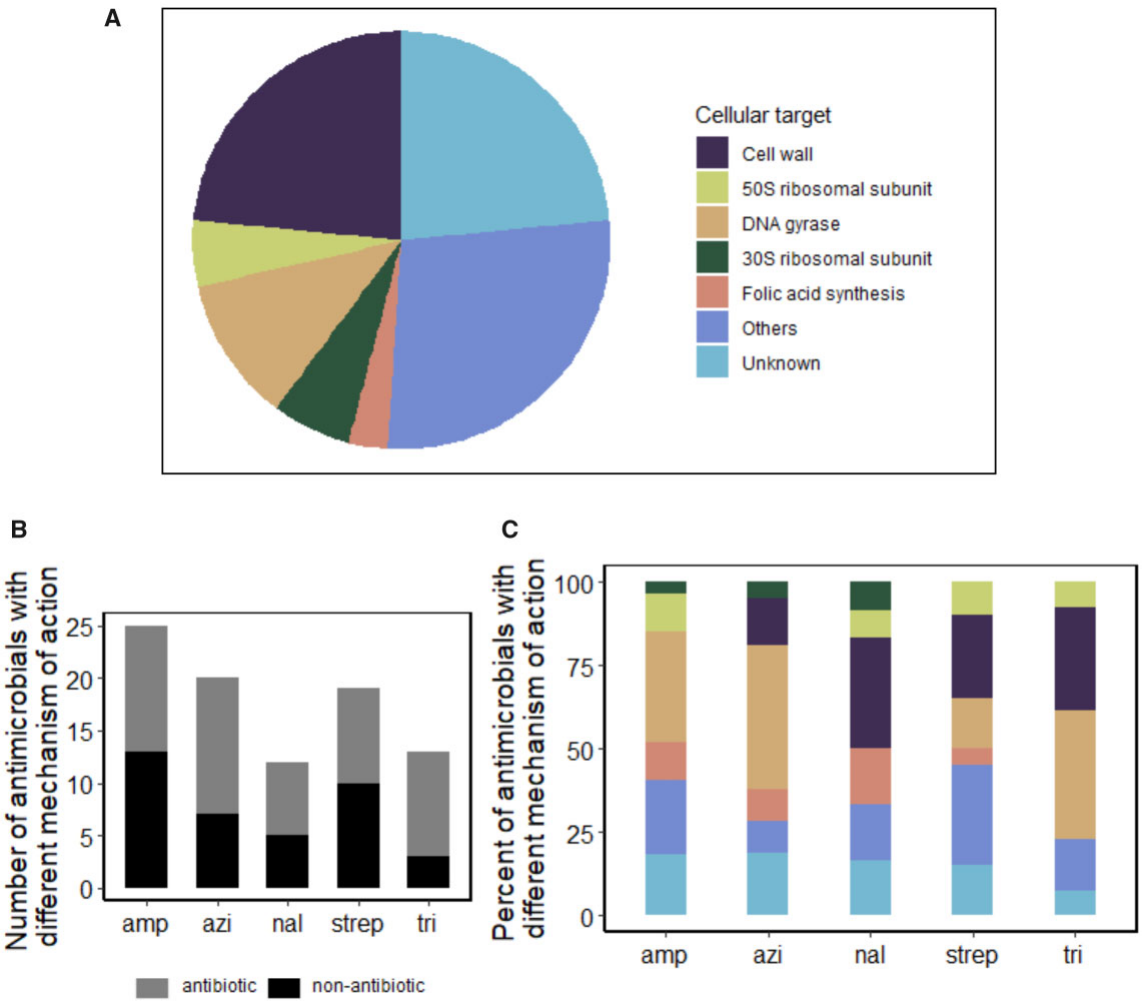
The majority of antimicrobials on which viability evolved have a different mechanism of action than the antibiotics in the evolution environment. (*A*) Classification of antimicrobials in those 95 phenotyping environments where viability evolved de novo, according to their mechanism of action and cellular target molecule. Five out of seven categories are those defined by the mechanism of action of the five antibiotics in our five evolution environments. The sixth category (“other”) includes antimicrobials where the mechanism of action is known but distinct from that of any of our five focal antibiotics. The seventh category comprises antimicrobials with an unknown mechanism of action. (*B*) Number of antimicrobials on which viability evolved de novo, and whose mechanism is not shared (vertical axis) with that of the antibiotic in the respective evolution environment (horizontal axis). For each evolution environment on the horizontal axis, these antimicrobials are further subdivided according to whether they are antibiotics (gray) or nonantibiotics (black). (*C*) Classification of novel traits from (*B*) based on the categorization of the phenotyping environment from (*A*), for each of the evolution environments (horizontal axis). The data show that in every evolution environment, viability evolved on antimicrobials whose mechanism of action falls into multiple categories different from that of the antibiotic in the evolution environment.

To test the hypothesis that resistance to most inhibitors is collateral resistance, we classified the 95 inhibitors on which viability evolved into 7 functional categories (fig. 3*A*). The first of them comprises ampicillin-like antimicrobials, which target the bacterial cell wall, and include antibiotics like amoxicillin and penicillin. Second, azithromycin-like antimicrobials target the 50S ribosomal subunit, and include antibiotics like chloramphenicol. Third, nalidixic acid-like antimicrobials target DNA gyrase, and include antimicrobials like norfloxacin. Fourth, streptomycin-like antimicrobials, such as amikacin, affect the 30S ribosomal subunit. Fifth, trimethoprim-like antimicrobials, such as azathioprine, target nucleotide biosynthesis. The sixth and largest class of antimicrobials (27 environments, category “others”) inhibit growth through a mechanism different from any one of the five antibiotics in the evolution environments. An example is the anticancer drug bleomycin, which primarily targets the furanose rings of DNA (Campbell et al. 2019). Seventh and finally, 23 of 95 environments inhibit bacterial growth by an unknown mechanism (fig. 3*A*).

We next determined for every antibiotic in our evolution environment the percentage of antimicrobial molecules 1) on which viability evolved, and 2) that have a different mode of action than the antibiotic in the evolution environment (fig. 3*B*). We found that the majority of antimicrobials on which viability evolved have a different mechanism of action (supplementary fig. S5, Supplementary Material online). For example, clones evolved on azithromycin evolved viability on 22 antimicrobials, and 91% of them (20 of 22) have a cellular target different from that of azithromycin. Only the antimicrobials tylosin and josamycin target the 50S ribosomal subunit like azithromycin does (fig. 3*B*). Similarly, 73% of antimicrobials (25 of 34) on which ampicillin clones acquired viability affect cells through a mechanism different from that of ampicillin. The corresponding numbers for clones evolved on nalidixic acid, streptomycin, and trimethoprim are 42% (5 of 12), 53% (10 of 19), and 23% (3 of 13), respectively.

Two of the five antibiotics that we used for experimental evolution have distinct cellular targets (fig. 1*B*), but they affect the same biological process. That is, streptomycin targets the 30S ribosomal subunit, azithromycin targets the 50S ribosomal subunit (Kapoor et al. 2017), but both antibiotics affect the process of protein synthesis. This observation raised the question whether all antimicrobials on which azithromycin- or streptomycin-evolved clones acquired viability may affect protein synthesis, even though they target a different molecule. However, this is not the case, because these antimicrobials fall into all seven categories (fig. 3*C*).

In sum, the antimicrobials on which viability evolves de novo act through a broad diversity of mechanisms, which are generally different from the mechanism of action of the antibiotic in the respective evolution environment.

### Novel Traits Arise through Pleiotropic Mutations

Genomic mutations can produce any one novel trait in one of two principal ways. First, a mutation may not affect resistance to the antibiotic in the evolution environment, but produce the novel trait. Such a mutation would be neutral or perhaps even mildly deleterious in the evolution environment, because it does not affect the focal antibiotic resistance trait. In other words, in this scenario, different mutations affect different traits, and whereas some mutations affect the primary resistance trait, others will help produce novel traits. A second possibility is that the same mutation that confers resistance to the antibiotic in the evolution environment also confers viability in a phenotyping environment. In this scenario, individual mutations show synergistic pleiotropy, that is, they have beneficial effects on multiple traits (Leiby and Marx 2014).

**Fig. 4.**
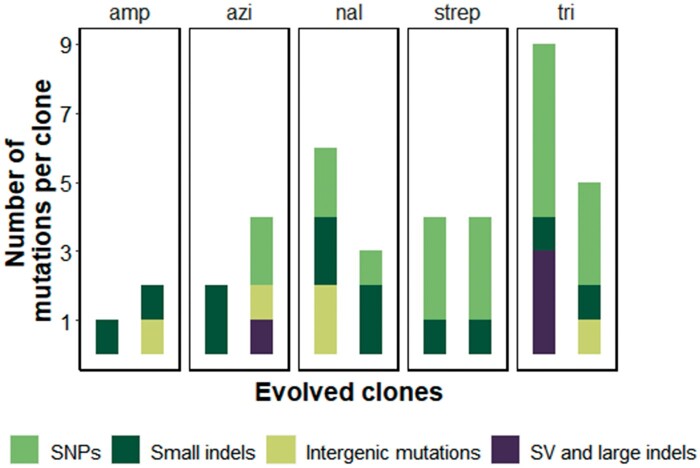
Evolved clones harbor few genomic mutations. Summary of all genomic mutations observed in the ten evolved clones. Each of the five panels contains data from the two evolved clones isolated from one of the five evolution environments, as indicated on top of each panel. We classified all observed genomic mutations into four categories. SNPs were the most common type of mutations. Specifically, we observed 18 SNPs in total, followed by 12 small indels (insertions or deletions smaller than 50 bp). We observed four small insertions (smaller than 50 bp), and one SNP in intergenic regions, that is, regions that do not encode a protein but might be involved in regulation. We also observed five instances of structural variants (SVs) or large indels (insertions or deletions larger than 50 bp). Ten out of the 12 observed insertions were mediated by IS elements.

To distinguish between these scenarios, we sequenced the genomes of our evolved clones. In total, all sequenced clones showed only 40 genomic mutations (fig. 4 and supplementary table S6, Supplementary Material online). Individual evolved clones harbored only between a total of 3 mutations for the 2 clones evolved in ampicillin (1 and 2 mutations per clone) and 14 mutations for clones evolved in trimethoprim (9 and 5 mutations per clone, fig. 4). The small number of genomic mutations we observed in our evolved clones is not consistent with the first, nonpleiotropic scenario. For example, even though the clones evolved on ampicillin harbored only 1 and 2 mutations each, they had become viable in 34 new environments. Thus, at least some of the mutations must be responsible for viability in more than one environment. In sum, the accumulation of nonpleiotropic mutations is not a likely cause for the evolution of novel traits.

To identify individual mutations with potentially pleiotropic effects, we first asked whether any mutations occurred in genes known to confer multidrug resistance. We found four instances of potentially multidrug-resistant mutations in four different evolved clones. First, one of the nalidixic acid clones harbored an insertion of 2 bp in the gene *mprA*. This gene encodes a transcriptional repressor of the antibiotic stress response (Lomovskaya et al. 1995). Loss-of-function mutations in *mprA* have been reported after evolution in antibiotics, and are known to confer resistance to antibiotics that target DNA gyrase, the 50S ribosomal subunit, and other macromolecules inside the cell (Lázár et al. 2014; Podnecky et al. 2018). Second, the gene encoding the transcriptional regulator Rob showed a nonsynonymous substitution in one of the azithromycin clones. Rob regulates the expression of *mar* complex that codes for a multidrug efflux pump, as well as of the *soxRS* regulon, which responds to oxidative stress (Keseler et al. 2017). Overexpression of Rob has been linked with increased resistance to multiple antibiotics (Ariza et al. 1995; Bennik et al. 2000). Third and fourth, both trimethoprim-evolved clones harbored a mutation in the *phoQ* gene, which encodes a protein that is a part of two-component regulatory system involved in acid stress tolerance (Zwir et al. 2005). Mutations in *phoQ* can result in resistance against antibiotics that target cell-wall synthesis, protein synthesis, as well as folic acid synthesis (Lázár et al. 2014).

The majority of clones did not harbor mutations known to confer resistance to multiple antimicrobials. We thus next looked for mutations in genes known to confer resistance against the antibiotic in the evolution environment. We reasoned that such mutations are strongly selected for during evolution, and are thus *a priori* good candidates to be causal for novel traits.

In general, mutations can confer resistance against a particular antibiotic in multiple ways (Blair et al. 2015). They can modify the cellular target of the antibiotic, allow enzymes to cleave the antibiotic, or upregulate efflux pumps that help export the antibiotic. Most of our clones harbored mutations in a cellular target protein of the relevant antibiotic, or in a protein interacting with that target. Specifically, one clone evolved on azithromycin harbored a 1 bp insertion in the gene *rpmH*, which encodes protein L34 of the 50S ribosomal subunit that is the cellular target of azithromycin (Keseler et al. 2017). The other azithromycin clone harbored a single nucleotide polymorphism (SNP) downstream of gene *rrlH*. This gene encodes the 23S ribosomal RNA, which is a part of 50S ribosomal subunit (Keseler et al. 2017). In both clones evolved on nalidixic acid, the same amino acid had mutated (D87Y in one clone and D87G in the other) in the *gyrA* gene, which encodes the DNA gyrase target of nalidixic acid. More generally, mutations at this locus confer resistance to quinolone antibiotics (Bhatnagar and Wong 2019). Both clones evolved on streptomycin showed the same two potentially resistance-conferring mutations. The first mutation is an SNP (I828N) in the gene *infB*, which encodes the translation initiation factor IF-2 that interacts closely with the 30S ribosomal subunit, the cellular target of streptomycin (Marzi et al. 2003; Caserta et al. 2006). The second mutation is a 25 bp deletion in gene *rsmG*, which encodes a 16S rRNA m(7)G527 methyltransferase. 16S rRNA is an integral part of the 30S ribosomal subunit (Okamoto et al. 2007). One of the clones evolved on trimethoprim showed two SNPs (A26T and W30G) in the gene *folA*, which encodes dihydrofolate reductase, the target of trimethoprim (Toprak et al. 2012). The other clone also harbored the W30R SNP in *folA* along with a SNP in *folE* (S206P) that encodes GTP cyclohydrolase 1. GTP cyclohydrolase 1 catalyzes the first step in the biosynthesis of tetrahydrofolate (Keseler et al. 2017). Finally, one of the ampicillin clones had a 1 bp insertion in the gene *frdD* which encodes one of two integral membrane proteins in the four subunit fumarate reductase complex. Mutation of *frdD* has been implicated in the resistance for ampicillin (Li et al. 2019). Although the mechanism is unknown, *frdD* is thought to be involved in a metabolic pathway that confers resistance to ampicillin (Li et al. 2019). The other ampicillin clone harbored a single small insertion in the gene *fimE* that regulates the expression of type I fimbrae. This gene has not been linked to cell-wall synthesis. However, cells evolved in the β-lactam antibiotic mecillinam have been reported to harbor a mutation in *fimE* (Podnecky et al. 2018). In sum, nine out of ten clones (all but one clone evolved on ampicillin) harbored at least one mutation in a gene that either encodes a cellular target of the relevant antibiotic, or a protein interacting with that target.

Genes affected by these mutations may also have pleiotropic effects on novel traits, for example, if these genes participate in more than one cellular process, such as protein and DNA synthesis (Webber et al. 2013). Two of the mutated genes—*gyrA* and *infB—*serve as examples. The DNA gyrase encoded by *gyrA* experienced the mutation D87G (changing aspartatic acid into glycine at the 87th position) in one nalidixic acid clone, and D87Y (changing aspartic acid into tyrosine) in the other nalidixic acid clone. The mutation is known to increase resistance against β-lactams and aminoglycosides, probably by affecting the supercoiling of DNA, which can in turn modify global gene expression patterns (Webber et al. 2013; Lázár et al. 2014). Second, the translation factor gene *infB* became mutated in both streptomycin-evolved clones. Mutations in this gene confer resistance against macrolide antibiotics that target protein synthesis through the 50S ribosomal subunit (Binh et al. 2014).

In sum, the large number of novel traits we identified, together with the small number of mutations per evolved clone, and the known pleiotropic nature of some of these mutations, suggest that pleiotropy is important for the evolution of novel traits.

## Discussion

Our work shows that multiple novel traits that are not the primary target of natural selection arise during short evolution experiments comprising no more than 250 generations. Specifically, our evolving populations became viable in between 16 and 34 out of 95 environments where their ancestor had not been viable. These environments harbored a wide variety of antimicrobial agents, including antibiotics, organic and inorganic salts, nucleotide analogs, pyridine derivatives, and surfactants. Most importantly, none of these molecules were present in the environment during experimental evolution. Our work also shows that phenotyping evolving populations in multiple environments can be crucial to identify novel traits that may become not just adaptive but essential for survival in some environments.

The origin of new microbial traits that are immediately beneficial in their environment of origin has been studied by others. For example, after ∼150 generations of laboratory evolution in multiple environments containing one of ∼100 different carbon sources, *P.aeruginosa* populations gained the ability to grow on eight new carbon sources (Toll-Riera et al. 2016). Because each environment contained only a single carbon source, the ability to metabolize this carbon source had large adaptive value in this environment. Similarly, the ability to grow on citrate evolved in 1 of 12 *E.coli* populations during a long-term evolution experiment (Blount et al. 2008, 2012). In this experiment, glucose was the primary carbon source, but the medium also contained citrate that the bacteria can normally not utilize. The ability to grow on citrate was thus immediately beneficial. In the same long-term evolution experiment, evolving populations also acquired the ability to grow on a variety of carbon sources that bacteria can excrete into the medium, including succinate, malate, aspartate, and fumarate (Leiby and Marx 2014). In sum, microbes readily evolve novel traits in environments where these traits are adaptive.

In principle, a novel trait whose origin requires only a single mutation may readily originate in evolution, regardless of whether it confers an immediate fitness advantage or not. The traits we studied, which involve viability on antimicrobial agents that are not present in the environment in which a population evolves, are such traits. Isolated examples from previous studies hint that traits without immediate benefits can indeed readily emerge during experimental evolution. For instance, clones isolated from *E.coli* populations evolved in the presence of the cell-wall targeting antibiotic cefepime become resistant to more than ten other antibiotics, including kanamycin, which targets protein synthesis, and chloramphenicol, which targets lipopolysaccharides (Imamovic and Sommer 2013). The same study evolved *E. coli* on 1 of 23 different antibiotics, and determined the resistance profiles of isolated clones on the remaining 22 antibiotics. Seventeen evolved clones showed improved resistance to at least one antibiotic that did not share the same mechanism of action as the focal antibiotic (Imamovic and Sommer 2013). Other studies have also reported a collateral increase or decrease in resistance after evolution in an environment containing antibiotics, but their primary focus was to establish collateral sensitivity or resistance profiles for clinically relevant antibiotics (Lázár et al. 2014; Munck et al. 2014; Yen and Papin 2017; Podnecky et al. 2018). As a result, they used only few phenotyping environments. In addition, they phenotyped evolved clones chosen at random, and collateral resistance profiles often varied between two clones from the same evolution environment (Lázár et al. 2014; Podnecky et al. 2018).

In contrast, we isolated clones that best represent the central tendency of a population’s viability in its evolution environment (fig. 1*C*). This means that our results are more likely to reflect typical viability patterns for any one evolved population. In addition, to score a trait as novel, we required that both evolved clones had become viable in a given phenotyping environment where none of two ancestral clones had been viable. Even with this stringent criterion, we observed that viability readily evolved on multiple clinically relevant antibiotics (supplementary table S3, Supplementary Material online). A particularly alarming observation is that all evolved clones became viable on the three clinically relevant antibiotics norfloxacin, cefuroxime, and cefotaxime (fig. 2*B*), even though none of these antibiotics occurred in the evolution environment. In addition, we showed that viability evolved not just on antibiotics different from those in the evolution environment, but also on multiple nonantibiotic growth inhibitors.

Viability on these nonantibiotic compounds is relevant clinically, as the example of antibiotic resistance breakers (ARBs) illustrates. ARBs are potential alternatives to conventional antibiotics, to which many pathogens have become resistant (Brown 2015; Czaplewski et al. 2016; Lagadinou et al. 2020). They are repurposed drugs and nutraceuticals that can be administered alone or together with an antibiotic to delay resistance evolution. It is clinically important to know the cross-resistance profiles of these and other antimicrobials. For example, our ampicillin-resistant clones also evolved viability on lawsone, which is a plant naphthoquinone and a candidate antimicrobial against multidrug resistance pathogens (Rahmoun et al. 2012; Soliman et al. 2017). Similarly, streptomycin clones evolved viability on chlorpromazine, which is typically used to treat psychotic disorders such as schizophrenia, but is also effective against biofilm forming bacteria (Tozar et al. 2019), including *Mycobacterium tuberculosis* (Hollister et al. 1960). Experiments like ours can help determine collateral resistance profiles of nonantibiotic antimicrobials.

The short duration of our evolution experiments is an advantage in identifying candidate mutations that help create novel traits, because only few mutations accumulated in our evolved lineages. Specifically, our evolved clones showed many fewer mutations (1–9) per clone than novel traits (16–34). Thus, at least some of the novel traits must have been caused by mutations that affect multiple traits. An abundance of such synergistically pleiotropic mutations is consistent with previous work. For example, in a long-term experiment that evolved *E. coli* in a glucose environment, cells improved their ability to grow on other sugars that they did not encounter during the experiment (Travisano and Lenski 1996; Leiby and Marx 2014). Likewise, mutations that help improve the growth of *E. coli* on one antibiotic do the same for other antibiotics (Lázár et al. 2014; Podnecky et al. 2018).

We observed seven-candidate pleiotropic mutations. They occurred in five different genes, namely *mprA, rob, phoQ, gyrA*, and *infB*. We speculate that these mutations helped *E.coli* become viable on at least some of the multiple antimicrobial agents we studied (supplementary table S5, Supplementary Material online). Three of these five genes, namely *mprA, rob*, and *phoQ*, encode regulators that can modulate the expression of many genes, including genes encoding multidrug efflux pumps (Ariza et al. 1995; Lomovskaya et al. 1995; Bennik et al. 2000; Zwir et al. 2005; Lázár et al. 2014; Keseler et al. 2017; Podnecky et al. 2018). An important task for future work is to confirm the role of candidate mutations for specific resistance phenotypes by engineering them into the ancestral strain.

Interactions between the few genomic mutations we observed may be important to produce novel traits. A possible example involves one of our ampicillin-evolved clones, which only harbored a single genomic mutation, a 4 bp insertion in the *fimE* region. Ampicillin-evolved clones displayed 34 novel traits, and shared 20 of these traits with clones evolved on nalidixic acid, one of which also harbored a 4 bp insertion in the *fimE* region (in addition to other mutations). This leaves 14 traits that cannot be accounted for by the shared mutation. This difference between the evolved novel traits might be explained by the interaction between the *fimE* mutation and the unique evolved genetic background of nalidixic acid-evolved clones. To prove the importance of such interactions, it would be necessary to transfer multiple alleles and their combinations into the ancestral strain background.

In recent years it has become increasingly clear that inherited nongenetic variation may play a role in adaptive evolution (Wolff 1978; Cubas et al. 1999; Sollars et al. 2003; Rassoulzadegan et al. 2006; Calhoun et al. 2014). Such variation may affect RNA and protein expression levels, as well as the concentration and the activity of other biomolecules (Day 2016). In environments containing an antibiotic, for example, bacterial populations can produce persister cells that escape death by ceasing to grow. When the antibiotic is removed from the environment, such persisters resume growth and produce both persister and nonpersister offspring (Bigger 1944). Nongenetic mechanisms of inheritance have been implicated in the formation of the persistence phenotype (Day 2016). In our experiments, viability on some antimicrobials may have evolved through newly acquired persistence.

In addition to nongenetic inheritance, phenotypic plasticity can in principle also cause viability on some antimicrobials on which the ancestor was inviable. By requiring that no ancestral clone displays a novel trait, whereas two independently isolated evolved clones display the trait, we aimed to reduce the likelihood of being misled by nongenetic variation and phenotypic plasticity. Also, many of our novel traits involve antimicrobial agents with the same mechanism of action as our five primary antibiotics, for which we observe genomic resistance mutations. Although this observation suggests that our novel traits are genetic in origin, we cannot completely exclude a nongenetic origin for some of them. One candidate example illustrates this point. Whole-genome sequencing showed that the two clones evolved in streptomycin harbored identical genomic mutations. Both clones were viable in 19 phenotyping environments where the ancestral clones were inviable (novel traits for “strep” in fig. 2*A* andsupplementary fig. S7, Supplementary Material online). One of the two clones was additionally viable in 11 phenotyping environments, whereas the other clone was viable in 4 phenotyping environments (supplementary fig. S7, Supplementary Material online; the viability in these additional (11 + 4) environments does not meet our criterion of a novel trait, which requires that both clones have become viable in a new environment) Because the clones were genetically identical, nongenetic causes, including possibly phenotypic plasticity, may be responsible for the evolution of viability on some additional antimicrobials. To determine the role of nongenetic variation and phenotypic plasticity would have required us to phenotype the same clone in the same environment multiple times. Performing the required assays for all 236 environments would have been prohibitive in terms of both times and cost. This remains a limitation of our work, and an important task for future experiments.

Although Biolog phenotyping microarrays allowed us to determine the phenotype of evolved clones in hundreds of environments, they may not be suitable to detect all kinds of novel traits. A case in point is a study of metabolic traits in *E. coli* after 50,000 generations of experimental evolution (Leiby and Marx 2014). The authors discovered that growth in Biolog plates, which are incubated without shaking, might not reflect growth in the well-mixed conditions typically used in experimental evolution, such as a shaken conical flask. Also, the Biolog phenotyping assay rests on measuring cell respiration, which can take place independently of cellular growth in some environments. However, these limitations are not likely to affect our central observations. First, we used PM11-20 Biolog phenotypic microarrays (Bochner 2009; Bochner et al. 2001) rather than GNII or GNIII microarrays that are frequently used for metabolic assays, and for which this problem has been described (Leiby and Marx 2014). GNII or GNIII arrays contain a minimal medium supplemented with a single carbon source, whereas PM11-20 arrays use the rich growth medium supplemented with different antimicrobial agents. To our knowledge, respiration is generally accompanied by growth in the PM11-20 environment. Second, we compared the phenotypes of ancestral and evolved clones that are grown under identical conditions in the Biolog environment. Unless ancestral and evolved clones are differently affected by this environment, the observation that evolved clones are viable in more antimicrobial environments than their ancestors is still valid. Its validity depends of course on the environments in which one measures viability, but this caveat applies to any comparable experiment.

Our work provides exciting directions for future research on the role of the environment and its complexity in the origin of novel traits. Even though our evolution environments were very simple, containing only a single antibiotic each, viability on multiple antimicrobials emerged, including antimicrobials whose mechanism of action differed from that of the focal environment. Natural environments are much more complex. Would life in such complex environments bring forth even more novel traits without immediate benefits? If so, we can expect naturally occurring microbes to harbor a wealth of such traits. Some of them may get lost again before they become beneficial. Others will remain unseen until the right environment brings out their benefits. Such traits may thus constitute an ever-changing reservoir of latent traits that can facilitate adaptive evolution in the right circumstances.

## Materials and Methods

### Bacterial Strain, Media, and Antibiotics

We used a previously described derivative of *E. coli* strain K12 MG1655 (MR^S^) for experimental evolution (Sprouffske et al. 2018). Henceforth we refer to this strain as the ancestor. For all our experiments, we used five different antibiotics, namely trimethoprim, azithromycin, streptomycin, ampicillin, and nalidixic acid (all obtained from Sigma). We chose these antibiotics because each targets a different cellular process (fig. 1*B*). We prepared stock solutions of each antibiotic and stored them at −20 °C without any exposure to light (supplementary table S8, Supplementary Material online). We used LB broth (Sigma) supplemented with the relevant antibiotic for all pilot and evolution experiments. To prepare a glycerol stock of our ancestral strain, we picked a colony of this ancestor from an LB agar plate, and inoculated it in 100 ml LB in a conical flask without any antibiotic.

We incubated the flask at 37 °C with shaking at 220 rpm (INFORS HT, Switzerland). After 20 h of growth, we mixed the 800 µl of bacterial culture with 200 µl of 15% glycerol (v/v) in a screw-capped tube and stored it at −80 °C. We call this the ancestral glycerol stock. Before experimental evolution, we determined the IC_90_ for each antibiotic, that is, the lowest antibiotic concentration that causes a 90% reduction in a culture’s optical density at 600 nm (OD_600_) after 24 h of growth of the ancestral strain, compared with growth in media without any antibiotic (Imamovic and Sommer 2013). For IC_90_ estimations, we revived 10 µl of the ancestral glycerol stock in 3 ml LB for 20 h at 37 °C with shaking at 220 rpm (INFORS HT, Switzerland). For every antibiotic, we inoculated three separate wells of a 24-well plate (Corning, USA) with 4 µl of this revived culture in 2 ml of LB supplemented with the antibiotic. We incubated the plate at 37 °C (350 rpm, SI505, Stuart, UK), and measured the OD_600_ after 24 h of growth in a plate reader (Tecan, Infinite 200 PRO). Supplementary table S1, Supplementary Material online lists the IC_90_ for each of the five antibiotics. IC_90_ values that we determined are equal to or greater than the clinical breakpoints for *E. coli* suggested by the European Committee on Antimicrobial Susceptibility Testing (EUCAST 2021).

### Experimental Evolution

We prepared an ancestral culture by reviving 10 µl of the ancestral glycerol stock in 3 ml LB for 20 h at 37 °C with shaking at 220 rpm (INFORS HT, Switzerland). We established eight replicate populations in each of the five antibiotics by mixing 4 µl of ancestral culture with 2 ml of LB supplemented with an antibiotic. We performed experimental evolution in 24-well plates with 2 ml of LB supplemented with an antibiotic. During experimental evolution, we transferred 4 µl of culture every day from evolving populations and incubated all cultures at 37 °C with shaking at 350 rpm (SI505, Stuart, UK). We increased the concentration of each antibiotic every other day until it had reached the IC_90_, at which point we terminated experimental evolution. We chose this procedure to minimize extinctions without allowing long periods of growth at any one antibiotic concentration. If the OD_600_ (Tecan, Infinite 200 PRO) had only reached a value between 0.2 and 0.3 after 20 h of incubation, we transferred 20 µl of inoculum instead of 4 µl to ensure survival of the population. This was necessary for two azi populations on the 16th day, and for four tri populations on the 19th, 20th, and 21st day of experimental evolution. We considered any growth below the threshold of 0.2 for OD_600_ as an extinction. We stored every day’s plates at 4 °C for 72 h. When a population became extinct, we used 20 µl of inoculum from the same replicate population of the previous day’s plate to resume evolution. We chose these growth thresholds based on pilot experiments which had shown that extinction rates are high for values of OD_600_ below 0.2, and moderate for values between 0.2 and 0.3.

Once per week, we streaked a sample of every population on LB agar plates, and inspected the sample visually for contamination after 20 h of incubation at 37 °C. After confirming purity, we prepared glycerol stocks and stored them at −80 °C. Only one instance of contamination occurred during experimental evolution. Specifically, one tri population became contaminated on the 13th day of evolution. We examined plated samples of the affected tri population from the preceding 3 days, and revived the population from the latest uncontaminated sample by reinoculating 20 µl of volume into fresh medium with trimethoprim. We note that none of the two representative tri clones we analyzed here stemmed from this population. At the end of experimental evolution, that is, when all populations could grow at the IC_90_ of the respective antibiotic, we prepared glycerol stocks of all the populations and stored them at −80 °C. Experimental evolution lasted for ∼108 to ∼215 generations, depending on the antibiotic (supplementary table S1, Supplementary Material online). We estimated the number of generations as the (base 2) logarithm of the dilution factor we had used for serial transfers (Bennett et al. 1992).

### Isolation of Representative Clones

We chose two representative clones from evolved populations for each antibiotic to examine novel traits and to sequence their genomes. To obtain these clones, we streaked a sample of a population’s glycerol stock on an LB agar plate, grew the sample overnight at 37 °C, and chose three colonies from the plate at random. We established a liquid culture from each colony in 2 ml LB without antibiotic, and allowed the culture to grow for 20 h at 37 °C in a shaking incubator (220 rpm, INFORS HT, Switzerland). We prepared a glycerol stock of the resulting culture and stored it at −80 °C. We then revived the glycerol stocks of all isolated clones, along with glycerol stocks of the eight replicate populations of each antibiotic environment. For this purpose, we inoculated 200 µl of LB with 4 µl of each glycerol stock in a 96-well plate (Thermo), and incubated the plate for 20 h at 37 °C in a shaking incubator (350 rpm, SI505, Stuart, UK). We transferred 4 µl of each resulting culture into 200 µl of culture medium containing the last day evolution environment i.e. the IC_90_ of the respective antibiotic. We then measured the OD_600_ every 15 min during 24 h of growth for all the clones and for the whole populations (Tecan, Infinite 200 PRO). In this manner, for every antibiotic, we obtained 8 different whole-population growth trajectories and 24 growth trajectories for the clones isolated from the 8 populations. We then used the GrowthRates software (Hall et al. 2014) to determine growth rates for all cultures. We computed 95% CIs for the mean growth rate from the eight whole population growth trajectories for a given antibiotic environment, and identified those clones whose growth rate lay within the 95% CIs of the populations. We reasoned that these clones are the best representatives of the central tendency of the populations that evolved in the antibiotic. From this subset of representative clones, we randomly chose two clones for each environment for further analysis. In addition, we also plated the ancestral culture on an LB agar plate, allowed the colonies to grow for 22 h at 37 °C, and randomly selected two clones. We then inoculated all the evolved (2 clones × 5 antibiotics) and ancestral (2) clones, 12 clones in total, in 2 ml LB and allowed them to grow for 20 h at 37 °C at 220 rpm. We stored the cultures as glycerol stocks to be used for phenotypic assays and genomic DNA extraction.

### Novel Trait Assays

We assessed the evolution of novel traits in the evolved clones using ten phenotypic microarrays provided by Biolog (PM11-20, Biolog, CA, USA; Bochner et al. 2001). These microarrays consist of microwell plates that harbor preconfigured sets of antimicrobials, and use a tetrazolium dye as an indicator for cell respiration. With an increase in respiration, cells produce more and more NADH which reduces the tetrazolium dye to produce a purple color. The intensity of this color is an indication of respiration and can be measured spectrophotometrically. Each microwell in a Biolog plate contains 1 of 236 potentially bactericidal or bacteriostatic molecules, and each such molecule is supplied at 4 different concentrations in different wells (240 molecules as per the manufacturer, but see supplementary text S9, Supplementary Material online for details). The actual concentration range of each molecule varies among molecules, and is proprietary information. Inhibitory substances in the environment include, but are not limited to, antibiotics, organic, and inorganic salts, nucleotide analogs, pyridine derivatives, and surfactants. We gathered information on the mechanism of action for these molecules from DrugBank, PubChem, and original research articles (Wishart et al. 2018; Kim et al. 2021). For 38 out of the 236 molecules, we could not find any relevant information.

To find out whether the clones we studied were able to grow in any one of these environments, we inoculated each evolved and ancestral clone in 2 ml LB with 4 µl of the glycerol stock, and allowed the resulting culture to grow for 20 h at 37 °C in an incubating shaker (220 rpm, INFORS HT, Switzerland). We then diluted the culture exactly according to the plate manufacturer’s protocol, and used it to inoculate one set of ten microarray plates (PM11-20). We incubated the plates at 37 °C for 48 h (SI505, Stuart, UK), measuring the OD_600_ immediately after inoculation (0 h), and after 48 h of growth (Tecan, Infinite 200 PRO). We used the initial (0 h) reading as a blank and subtracted it from the measured OD_600_ at 48 h to account for the instrument and inoculum background. We considered a growth phenotype as a novel trait if both ancestral clones showed an OD_600_ below 0.3 after 48 h, whereas both evolved clones from the same antibiotic showed an OD_600_ above 0.3 after 48 h. This threshold of growth is motivated by the observation that inoculating 230 of the Biolog environments yields an OD_600_ below 0.3 immediately after inoculation, that is, at 0 h (supplementary fig. S10, Supplementary Material online). Our central observation that novel traits are widespread is robust to this threshold choice (supplementary fig. S4, Supplementary Material online).

Because the ancestor was viable on the first three concentrations of most of the antimicrobials that define individual phenotyping environments (supplementary fig. S11, Supplementary Material online), the respective environments did not present an opportunity for the evolution of novel traits. We thus henceforth considered only the highest concentration of the antimicrobial molecules that define the 236 environments. Because both ancestral clones were unable to grow in only 95 of the 236 environments at the highest concentration of the respective antimicrobial molecule (supplementary fig. S11, Supplementary Material online), the maximally possible number of novel viability traits is 95.

### Whole-Genome Sequencing of Ancestral and Evolved Clones

We extracted the genomic DNA of all evolved and ancestral clones using the DNeasy Blood and tissue kit from Qiagen (catalog no. 69504). For this purpose, we inoculated 5 ml of LB without antibiotic using 4 µl of glycerol stock of each clone, and allowed the resulting culture to grow for ∼16 h at 37 °C with shaking at 220 rpm (INFORS HT, Switzerland). We harvested ∼2 × 10^9^ cells from this culture by centrifugating at 7,500 rpm for 10 min (Eppendorf 5810/5810 R). We extracted DNA from the harvested cells according to the kit’s protocol and quantified the purity of the extracted genomic DNA with a Qubit fluorometer (Thermo Fisher Scientific), as well as through agarose gel electrophoresis. We stored the extracted DNA at −20 °C. The whole genome of each clone was sequenced using the Illumina HiSeq (Illumina, CA, USA) at MicrobesNG (Oxford, UK) to a minimum coverage of 30-fold per clone. We obtained the trimmed reads as fastq files from MicrobesNG and identified mutations using the breseq pipeline v0.35 with default parameters (Deatherage and Barrick 2014). We only considered those mutations that were not present in the ancestor as novel mutations. We observed 40 such mutations across the ten evolved clones. Using the Integrative Genomics Viewer (v2.9.2, Broad Institute, CA, USA) we visually confirmed each one of these mutations by comparing the reads from ancestral and evolved clones at that locus. We used curated descriptions on EcoCyc and references therein to annotate the function of each mutated gene (Keseler et al. 2017).

## Data Processing

We used R software (v3.5.2) to process the data and calculate descriptive statistics (fig. 1*C*).

## Supplementary Material

Supplementary data are available at *Molecular Biology and Evolution* online.

## Acknowledgments

This project has received funding from the European Research Council under Grant Agreement No. 739874. We would also like to acknowledge support by Swiss National Science Foundation grant 31003A_172887 as well as by the University Priority Research Program in Evolutionary Biology.

## Data Availability

The data underlying this article are available in the article and in its online supplementary material. Genome sequence data is available on request.

## Supplementary Material

msab341_Supplementary_Data

## Notes

The research was done at Department of Evolutionary Biology and Environmental Studies, University of Zurich, Zurich, Switzerland.
