# Supplementary material for: Multiple Novel Traits without Immediate Benefits Originate in Bacteria Evolving on Single Antibiotics
Source: Mol Biol Evol. 2021 Dec 3;39(1):msab341. doi: 10.1093/molbev/msab341 (PMC8789282; doi:10.1093/molbev/msab341)

Supplementary material

Table S1: IC_{90}, Number of days and estimated number of generations for evolution in each antibiotic. A pilot experiment had shown that bacterial tolerance for a daily increase in dosage was different for different antibiotics. For instance, we could increase the concentration of ampicillin twice as fast as that of trimethoprim without population extinction. This difference among antibiotic environments led to different durations of experimental evolution for different antibiotics.

Environment	IC_{90} in µg/ml	Starting concentration in µg/ml	Number of days	~ Number of generations
Ampicillin	8	0.25	12	108
Azithromycin	25.6	0.2	16	143
Nalidixic acid	64	0.25	19	170
Streptomycin	16	0.5	12	108
Trimethoprim	409.6	0.4	24	215

Table S2: Growth rates (per minute, column 3 and 7 from the left), corresponding doubling times (in minutes, column 4 and 8) and lag times (in minutes, column 5 and 9) for the eight evolved populations (column 2) and three clones (column 6) from each evolved population for the five antibiotics (column 1). Growth rates of the two clones that we chose for the novel trait assay, and for whole-genome sequencing are marked with '*'.

Antibiotic	Population	Growth rate	Doubling time	Lag time	Clone	Growth rate	Doubling time	Lag time
Ampicillin	1	0.021	33	37.9	I	0.01778	39	39.3
					II	0.01698	40.8	17.1
					III	0.01711	40.5	49.8
	2	0.01902	36.4	76.9	I	0.0184	37.7	73.1
					II	0.01869	37.1	17.4
					III	0.01745	39.7	52.6
	3	0.02231	31.1	40.9	I	0.01875	37	25
					II	0.01926	36	40.9
					III	0.0185	37.5	69.9
	4	0.0231	30	48.3	I	0.01676	41.3	28.5
					II	0.0177	39.2	33.9
					III	0.01717	40.4	87.1
	5	0.02244	30.9	42.3	I	0.02032	34.1	16.3
					II	*0.01951	35.5	25.7
					III	0.01833	37.8	54.8
	6	0.0289	24	34.5	I	0.02057	33.7	19
					II	0.02233	31	30.7
					III	*0.02624	26.4	24.3
	7	0.02466	28.1	35.1	I	0.01909	36.3	7.2
					II	0.02073	33.4	32.7
					III	0.02375	29.2	24.1
	8	0.02002	34.6	24.5	I	0.02012	34.4	50.2
					II	0.0151	45.9	251.2
					III	0.01881	36.9	13.9
Azithromycin	1	0.0131	52.9	45.3	I	0.01231	56.3	-6.1
					II	*0.0146	47.5	38.5
					III	0.01483	46.7	52.2
	2	0.01425	48.6	15.6	I	0.0164	42.3	47.2
					II	0.01413	49.1	23.5
					III	0.01684	41.2	51.3
	3	0.01619	42.8	118.7	I	0.01773	39.1	56.8
					II	*0.01625	42.7	99.3
					III	0.0173	40.1	107.4
	4	0.0109	63.6	87.5	I	0.00831	83.4	48.7
					II	0.00664	104.5	51.7
					III	0.00673	103	45.5
	5	0.01625	42.7	101.3	I	0.01265	54.8	54.1
					II	0.01275	54.4	67.9
					III	0.01476	47	50.3
	6	0.01956	35.4	80.9	I	0.01981	35	66.8
					II	0.01902	36.4	42.7
					III	0.01228	56.5	57.8
	7	0.01572	44.1	121.2	I	0.01063	65.2	118
					II	0.01085	63.9	136.4
					III	0.00998	69.5	147.9
	8	0.01013	68.4	29.1	I	0.01026	67.6	31.2

					II	0.01056	65.6	37.2
					III	0.01075	64.5	41.4
Nalidixic acid	1	0.01326	52.3	52.6	I	0.01638	42.3	27
					II	0.01163	59.6	33.8
					III	0.01129	61.4	72.8
	2	0.02968	23.4	55.3	I	*0.0189	36.7	29.8
					II	0.02352	29.5	44.9
					III	0.02761	25.1	49.6
	3	0.02933	23.6	54	I	0.02401	28.9	37.3
					II	*0.02028	34.2	79.1
					III	0.02231	31.1	36.7
	4	0.01856	37.3	30.1	I	0.02816	24.6	46.2
					II	0.03024	22.9	63
					III	0.02982	23.2	42.3
	5	0.01198	57.9	11.2	I	0.012	57.8	50.7
					II	0.01337	51.8	64.5
					III	0.0142	48.8	114.9
	6	0.02126	32.6	37.7	I	0.02038	34	42
					II	0.01984	34.9	4.9
					III	0.02008	34.5	45.1
	7	0.0216	32.1	38.4	I	0.02112	32.8	34.9
					II	0.01983	34.9	9.3
					III	0.01996	34.7	35.2
	8	0.0192	36.1	61.3	I	0.01703	40.7	68.4
					II	0.02351	29.5	66.4
					III	0.01676	41.4	90.5
Streptomycin	1	0.02297	30.2	5.7	I	0.02145	32.3	7.3
					II	0.02248	30.8	-1.9
					III	0.02268	30.6	23.9
	2	0.01915	36.2	75.7	I	*0.01904	36.4	92.1
					II	0.01655	41.9	55.7
					III	*0.01679	41.3	86.2
	3	0.01747	39.7	55.3	I	0.01844	37.6	117.2
					II	0.01781	38.9	48.8
					III	0.01905	36.4	103.3
	4	0.01895	36.6	25.6	I	0.01817	38.1	71.7
					II	0.01694	40.9	27.5
					III	0.01661	41.7	51.9
	5	0.01988	34.9	40.4	I	0.01512	45.9	38
					II	0.0176	39.4	19.1
					III	0.01548	44.8	19.6
	6	0.0179	38.7	49.2	I	0.01492	46.4	81.7
					II	0.01673	41.4	79.7
					III	0.01727	40.1	59.9
	7	0.01843	37.6	44.2	I	0.01894	36.6	59.8
					II	0.01867	37.1	22.9
					III	0.01635	42.4	48.1
	8	0.01811	38.3	72.2	I	0.0189	36.7	82.1
					II	0.01813	38.2	65.8
					III	0.01699	40.8	63.2
Trimethoprim	1	0.01123	61.7	39.5	I	*0.00966	71.7	42.4
					II	0.01048	66.2	29.6
					III	0.01332	52	63.5
	2	0.01782	38.9	37.6	I	0.01795	38.6	28.9
					II	0.01456	47.6	12
					III	0.01511	45.9	34.1
	3	0.00251	276.1	868.3	I	0.00523	132.6	122.6
					II	0.00335	207.1	117.3
					III	0.00295	235	39.2
	4	0.00692	100.1	245.3	I	0.00495	140.1	38.6

					II	0.00365	189.8	84.9
					III	0.00551	125.9	146.6
	5	0.00674	102.8	195.6	I	0.00834	83.1	482
					II	0.01959	35.4	75.8
					III	0.00604	114.8	267.8
	6	0.01404	49.4	10.8	I	0.01538	45.1	41.6
					II	0.01705	40.7	55.2
					III	0.01462	47.4	54.6
	7	0.00836	82.9	131.4	I	0.00494	140.4	298.3
					II	0.00605	114.6	279.2
					III	0.00631	109.9	77.2
	8	0.00782	88.6	54.2	I	0.00525	132	54.2
					II	*0.00764	90.7	45.4
					III	0.00596	116.3	49.2

13

Table S3: List of Biolog antimicrobials on which viability evolved after evolution on the antibiotic indicated in the top row.

	**Ampicillin**	**Azithromycin**	**Nalidixic acid**	**Streptomycin**	**Trimethoprim**
1	1-hydroxy pyridine-2-thione	5,7-Dichloro-8-hydroxyquinoline	1-hydroxy pyridine-2-thione	1-chloro-2,4-dinitrobenzene	Azathioprine
2	5,7-Dichloro-8-hydroxyquinoline	Cefotaxime	Azathioprine	1-hydroxy pyridine-2-thione	Cefotaxime
3	Azathioprine	Cefuroxime	Cefotaxime	2,2-Dipyridyl	Cefuroxime
4	Blasticidin S	Cinoxacin	Cefuroxime	4-aminopyridine	Cinoxacin
5	Cefotaxime	Ciprofloxacin	Cinoxacin	5,7-Dichloro-8-hydroxyquinoline	Cioxacillin
6	Cefuroxime	Diamide	Ciprofloxacin	Blasticidin S	Ciprofloxacin
7	Chlorpromazine	Enoxacin	Diamide	Cefotaxime	Diamide
8	Cinnamic acid	Fusaric acid	Enoxacin	Cefuroxime	Enoxacin
9	Cinoxacin	Harmane	Fusaric acid	Cioxacillin	Fusaric acid
10	Cloxacillin	Josamycin	Gallic acid	Diamide	L-Aspartic-B-hydroxamate
11	Ciprofloxacin	Lauryl sulfobetaine	Harmane	Fusaric acid	Norfloxacin
12	Diamide	Lomefloxacin	Lomefloxacin	Glycine hydroxamate	Ofloxacin
13	Enoxacin	Minocycline	Minocycline	Hygromycin B	Oxacillin
14	Fusaric acid	Nafcillin	Nafcillin	Lauryl sulfobetaine	Sodium tungstate
15	Gallic acid	Nalidixic acid	Nalidixic acid	Nafcillin	Thiamphenicol
16	Harmane	Norfloxacin	Norfloxacin	Norfloxacin	Trimethoprim
17	L-Aspartic-B-hydroxamate	Novobiocin	Novobiocin	Novobiocin	
18	Lauryl sulfobetaine	Ofloxacin	Ofloxacin	Oxacillin	
19	Lawsone	Sodium cyanate	Sodium selenite	Phenylarsine oxide	
20	Lomefloxacin	Sodium selenite	Trimethoprim	Thiamphenicol	
21	Nafcillin	Trimethoprim	Tylosin	Tylosin	
22	Nalidixic acid	Tylosin			
23	Norfloxacin				
24	Novobiocin				
25	Ofloxacin				
26	Oxacillin				
27	Sodium cyanate				
28	Sodium selenite				
29	Sodium tungstate				
30	Sodium-m-arsenite				
31	Thiamphenicol				
32	Trimethoprim				
33	Tylosin				
34	Vancomycin				

Figure S4: Number of novel traits (upper two panels) and number of novel traits evolved in the environments with antimicrobials that do not share the mechanism of action with the respective antibiotic treatment (lower two panels) for the five antibiotic environments (horizontal axis). For the left-hand side panels, we considered that growth had occurred when both clones from the same antibiotic environment showed OD_{600}>0.2, whereas both ancestral clones showed OD_{600}<0.2. For the right-hand side panels, we used the same criterion, but with an OD_{600} threshold of 0.4. Novel traits are widespread irrespective of the choice of threshold.

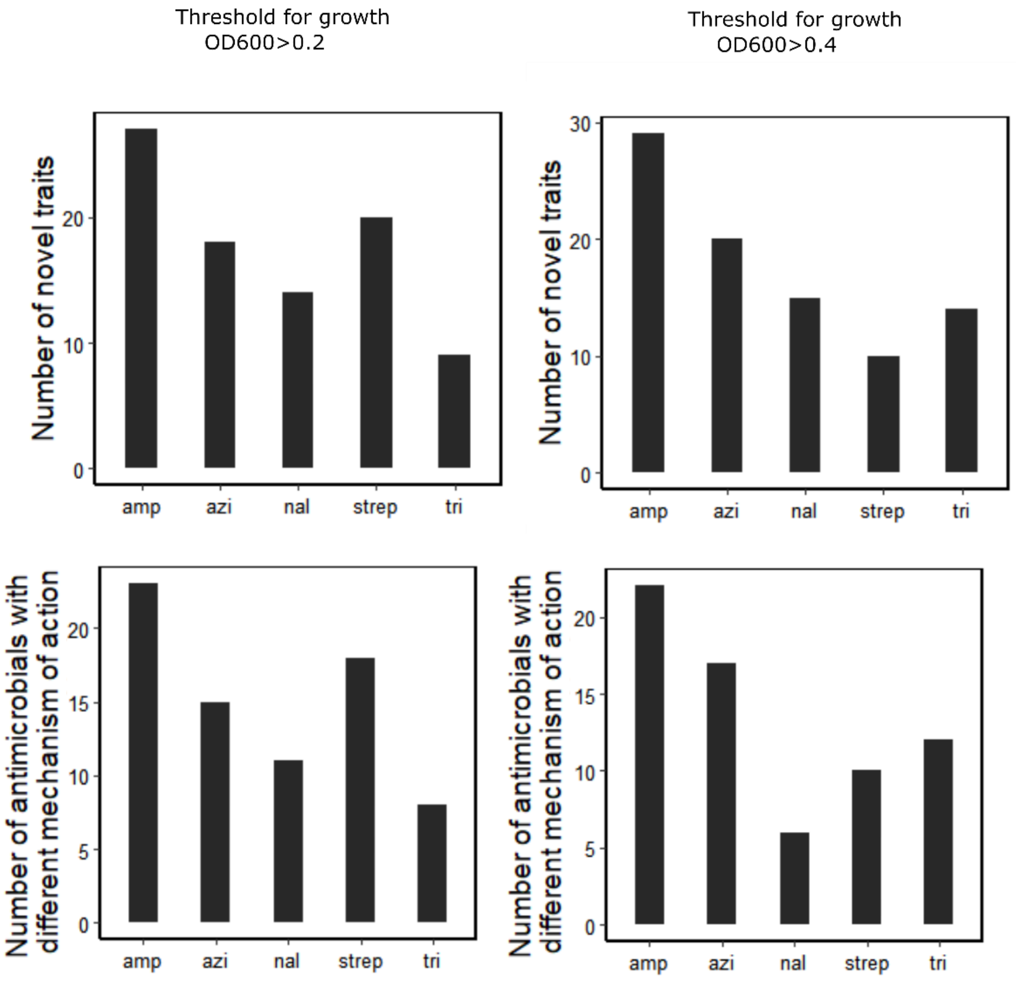

Figure S5: A. Number of all the novel traits after experimental evolution in each of the five antibiotics (same as figure 2A) and **B.** number of antimicrobials on which viability evolved *de novo*, and whose mechanism is not shared with that of the antibiotic in the respective evolution environment (data same as figure 3B) represented together for the direct comparison. Phenotyping environments are classified into two categories – one that contained an antibiotic (grey), and two that contained a non-antibiotic antimicrobial (black).

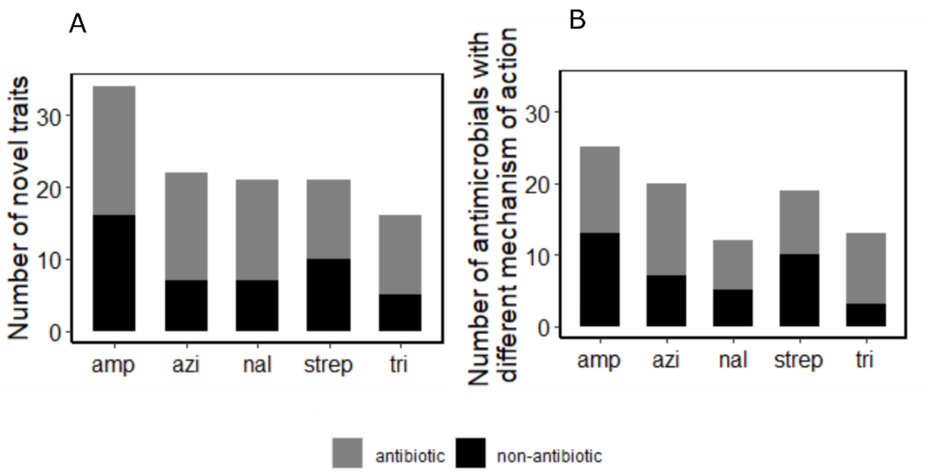

Table S6: All mutations (column 3 from the left) we observed in evolved clones. Candidate adaptive mutations (marked with '*') in the gene (column 4) that encodes the cellular target (Column 1) of the specified antibiotic (Column 2), a protein that directly interacts with that target, or a protein known to be involved in antibiotic resistance. All evolved clones, except one evolved on ampicillin, have at least one such candidate mutation. Candidate multi-drug resistance mutations are marked with a '+'.

**Cellular target**	**Environment and clone**	**Mutation**	**Gene**	**Mutation target**
Cell wall synthesis	Amp I	Small insertion (+4 bp)	fimE	Regulator for fimA that codes for the major subunit of type I fimbrae
	Amp II	*Small insertion (+T)	frdD	Fumarate reductase, integral membrane protein
		Small insertion (+4 bp)	Intergenic region between fimB and fimE	Both regulate fimA that codes for the major subunit of type I fimbrae
50S subunit in protein synthesis	Azi I	*Small insertion (+T)	rpmH	50S ribosomal subunit protein L34
		Small insertion (+9 bp)	sufC	Fe-S cluster scaffold complex subunit
	Azi II	*SNP(C > A)	downstream of rrlH	23S ribosomal RNA that is a part of the 50S subunit
		+SNP(D260N)	rob	A transcriptional regulator that is known to play a role in antibiotic and superoxide resistance
		Small insertion (+9 bp)	Intergenic region between ydeP and ydeQ	YdeP codes for putative oxidoreductase and YdeQ codes for putative fimbrial adhesin protein
		Large insertion (+1199 bp)	insH21	One of the putative copies of IS5 transposase and trans-activator
DNA gyrase	Nal I	+*SNP(D87Y)	gyrA	Subunit A of DNA gyrase and a known mutation in quinolone resistance
		SNP(L322F)	poxB	Pyruvate oxidase
		Small insertion (+4 bp)	ydiN	Putative transporter
		Small insertion (+9 bp)	Intergenic region between torY and micL	torY codes for cytochrome c quinol dehydrogenase and micL codes for small regulatory RNA
		Small insertion (+8 bp)	yiaM	2,3-diketo-L-gulonate:Na(+) symporter - membrane subunit
		Small insertion (+4 bp)	Intergenic region between fimB and fimE	Both regulate fimA that codes for the major subunit of type I fimbrae
	Nal II	+*SNP(D87G)	gyrA	Subunit A of DNA gyrase and a known mutation in quinolone resistance
		+Small indel (+CG)	mprA	DNA-binding transcriptional repressor
		Small insertion (+4 bp)	fimE	Regulator for fimA that codes for the major subunit of type I fimbrae
30S subunit in protein synthesis	Strep I	+*SNP(I828N)	infB	Translation initiation factor IF-2, which interacts closely with the 30S subunit
		*Small deletion (Δ25 bp)	rsmG	16S rRNA m(7)G527 methyltransferase. 16S rRNA is a part of the 30S subunit

		SNP(A249E)	yaiW	surface-exposed outer membrane lipoprotein
		SNP(S406N)	selA	selenocysteine synthase
	Strep II	[+]*SNP(I828N)	infB	Translation initiation factor IF-2, which interacts closely with the 30S subunit
		*Small deletion (Δ25 bp)	rsmG	16S rRNA m(7)G527 methyltransferase. 16S rRNA is a part of the 30S subunit
		SNP(A249E)	yaiW	surface-exposed outer membrane lipoprotein
		SNP(S406N)	selA	selenocysteine synthase
Dihydrofolate reductase in thymidine synthesis	Tri I	*SNP(W30R)	folA	Dihydrofolate reductase
		*SNP(S206P)	folE	GTP cyclohydrolase 1 catalyzes the first step in the biosynthesis of tetrahydrofolate
		[+]SNP(I207N)	phoQ	Sensory histidine kinase
		Large insertion (+1199 bp)	insH21	One of the putative copies of IS5 transposase and trans-activator
		Large insertion (+776 bp)	insB-5-insA-5	IS1 protein InsB and IS1 protein InsA
		Small insertion (+9bp)	yhhY	N-acetyltransferase
		Large deletion (Δ1759 bp)	Regions of yhiN and pitA	yhiN - putative oxidoreductase and pitA - metal phosphate:H+ symporter
		Synonymous SNP (D170D)	yibA	putative lyase containing HEAT-repeat
		SNP (D280G)	dnaA	chromosomal replication initiator protein
	Tri II	*SNP(A26T)	folA	Dihydrofolate reductase
		*SNP(W30G)	folA	Dihydrofolate reductase
		[+]SNP(I207N)	phoQ	Sensory histidine kinase
		Small deletion (Δ1 bp)	recE	Rac prophage, exonuclease VIII, ds DNA exonuclease, 5' --> 3' specific
		IS5 mediated large deletion (Δ16413 bp)	yhiN, pitA, uspB, uspA, dtpB, rsmJ, prlC, rlmJ, gor, dinQ, agrA, agrB, arsR, arsB, arsC and regions of yhiM and yhiS	yhiN - putative oxidoreductase, pit A - metal phosphate:H+ symporter, uspB - putative universal ethanol tolerance protein B, uspA - the regulator of protein B, dtpB - dipeptide/tripeptide:H+ symporter, rsmJ - 16S rRNA m2G1516 methyltransferase, prlC - oligopeptidase A, rlmJ - 23S rRNA m6A2030 methyltransferase, gor – glutathione reductase, dinQ – membrane toxin ,agrA - small RNA, agrB – small regulatory RNA, arsR - DNA-binding transcriptional repressor, arsB - arsenite/antimonite:H+ antiporter, arsC –arsenate reductase, yhiM – inner membrane protein, yhiS – Putative uncharacterized protein

Figure S7: The two clones (represented by green and red colours) evolved in streptomycin were viable in 19 phenotyping environments where both the ancestor clones were inviable. First clone evolved viability on additional 11 phenotyping environments while the second clone evolved viability on additional 4 phenotyping environments.

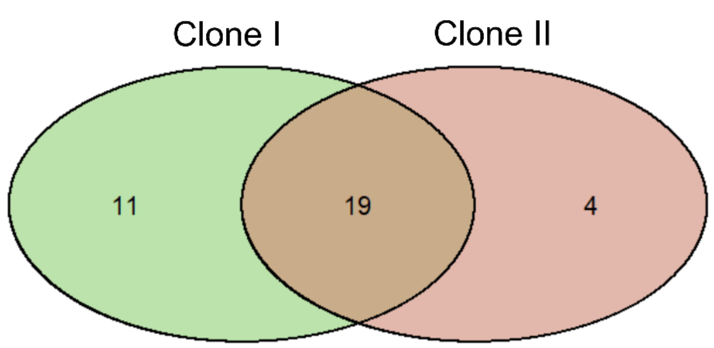

Table S8: Stock solutions of the antibiotics used for experimental evolution

Antibiotic	Solvent	Stock concentration
Trimethoprim	99% water + 1% glacial acetic acid	10 mg/ml
Azithromycin	Absolute ethanol	50 mg/ml
Streptomycin	Water	50 mg/ml
Ampicillin	99.5% water + 0.05% HCl	10 mg/ml
Nalidixic acid	99% water + 1% 10M NaOH	10 mg/ml

Supplementary text S9: Biolog plates contain 240 environments, as per the manufacturer's information (Biolog, USA) but four environments are present twice. These are environments containing carbenicillin, chloramphenicol, potassium tellurite, and polymyxin B. This means that all the ancestral and evolved clones were tested twice in these four environments. To determine whether a novel trait has evolved in these duplicate environments, we applied the same criteria as stated in Methods, but applied to four measurements rather than two. Specifically, for any duplicate environment, if all four wells harbouring ancestral clones (2 wells per clone) showed an OD_{600} below 0.3 after 48 hours of growth, and if all four wells harbouring evolved clones (2 wells per clone) showed an OD_{600} above 0.3, we considered a novel trait to have evolved.

Figure S10: We measured OD_{600} values at zero hours, i.e. immediately after inoculation, for both ancestral clones at the highest of four concentration for each of the 240 molecules that constitute Biolog environments. 460 of 480 (= 240 environments × 2 ancestral clones) OD_{600} measurements lay below the threshold of 0.3, and only 20 lay above the threshold. The horizontal axis shows OD_{600} at zero hours, and the vertical axis shows the frequency of Biolog environments with a particular OD_{600} value. The inset shows the distribution of all 480 measurement. The vertical dashed line indicates an OD_{600} of 0.3.

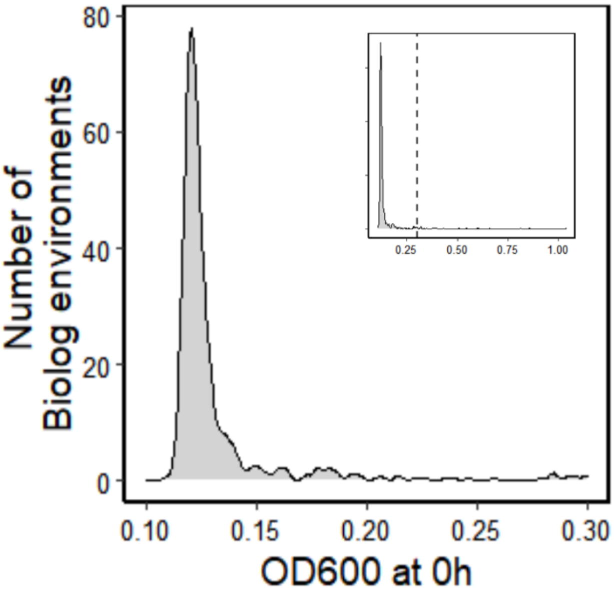

Figure S11: Number of Biolog environments, out of 236, in which at least one ancestral clone was able to grow, i.e., it showed an OD_{600} exceeding 0.3 after 48 hours of incubation. The horizontal axis indicates the four concentrations of each molecule defining an environment, where ‘1’ represents the lowest concentration, and ‘4’ represents the highest. Actual concentration values differ among environments and are proprietary information of Biolog Inc. (CA, USA).

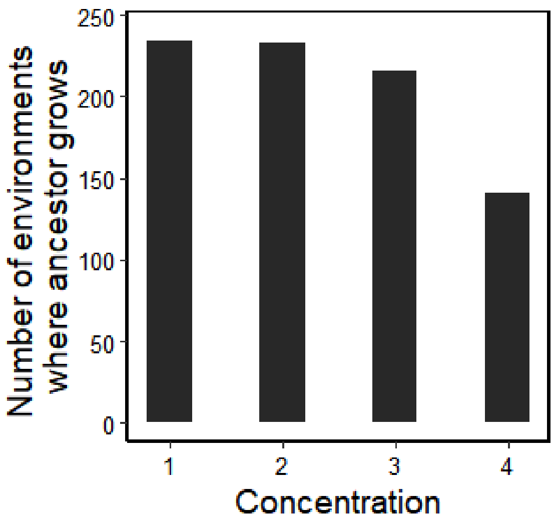

Supplement: msab341_Supplementary_Data [file msab341_supplementary_data.pdf]
